# Isoproterenol-induced changes in perfusion, function, energy metabolism and nitric oxide pathway: in vivo and ex vivo study in the rat heart

**DOI:** 10.1186/1532-429X-13-S1-P348

**Published:** 2011-02-02

**Authors:** Martine Desrois, Frank Kober, Carole Lan, Christiane Dalmasso, Mark A Cole, Kieran Clarke, Patrick J Cozzone, Monique Bernard

**Affiliations:** 1 CRMBM UMR CNRS 6612, Marseille, France; 2 University Laboratory of Physiology, University of Oxford, Oxford, UK

## Introduction

Calcium overload induced by chronic administration of the β-adrenoreceptor agonist, isoproterenol (IP), is used in animal to study the mechanisms of cardiac hypertrophy and failure associated with a sustained increase in circulating catecholamines.

## Purpose

Time-dependent changes in myocardial perfusion, morphologic and functional parameters were assessed in rats *in vivo* using cardiac MRI. Energy metabolism and nitric oxide (NO) pathway were evaluated in isolated perfused rat hearts following 7 days of treatment.

## Methods

Male Wistar rats were infused for 7 days via a subcutaneous minipump containing isoproterenol (5 mg.kg-1 body weight.day-1, IsoP group, n = 14) or vehicle (Control group, n = 14). Three MRI *in vivo* examinations were performed at day 1, 2 and 7 after pump implantation. A cine-MRI sequence (FOV 4 cm, slice thickness 2 mm, matrix size 128*128, TE=1.2ms, TR=5.1 ms) and a gradient-echo FAIR Look-Locker arterial spin labeling technique were used to determine cardiac function and perfusion at all stages. For *ex vivo* studies, isolated hearts were perfused with a physiological buffer for 28 min before freeze-clamping for biochemical assays. High energy phosphate compounds and intracellular pH were followed using P-31 MRS with simultaneous measurement of contractile function. The total creatine pool and malondialdehyde (MDA) content were measured by HPLC. NO pathway was evaluated by endothelial NO synthase expression and total nitrate concentration (NOx).

## Results

Left ventricular mass was increased in IsoP after 1 day and then maintained (p<0.05). Diastolic wall thickness was increased in IsoP with a peak at day 2 and a return to basal value at day 7 (p<0.05). Myocardial blood flow was increased at day 1 in IsoP group and returned to baseline values between days 1 and 2 (p<0.05). After 7 days of treatment, cardiac function in explanted hearts was reduced by 36 % in IsoP (p<0.05 vs Control). PCr/ATP ratio was lower (p<0.05) in IsoP (1.2±0.1) compared with Control (1.7±0.1). MDA content was higher in IsoP (p<0.05) compared with Control. eNOS expression and NOx were increased in IsoP (p<0.05 vs Control).

## Conclusions

IP increases myocardial perfusion and induces morphological changes in the heart within the first 24 hours of administration. In addition, cardiac hypertrophy and decreased cardiac function were associated with impaired phosphocreatine level, increased oxidative stress and up-regulation of the NO pathway. These results could lead to a better understanding of the initial steps involved in cardiac hypertrophy and failure.

